# Gaze-Stabilizing Central Vestibular Neurons Project Asymmetrically to Extraocular Motoneuron Pools

**DOI:** 10.1523/JNEUROSCI.1711-17.2017

**Published:** 2017-11-22

**Authors:** David Schoppik, Isaac H. Bianco, David A. Prober, Adam D. Douglass, Drew N. Robson, Jennifer M.B. Li, Joel S.F. Greenwood, Edward Soucy, Florian Engert, Alexander F. Schier

**Affiliations:** ^1^Departments of Otolaryngology, Neuroscience and Physiology, and the Neuroscience Institute, New York University School of Medicine, New York, New York 10016,; ^2^Department of Neuroscience, Physiology and Pharmacology, University College London, London WCE1 6BT, United Kingdom,; ^3^Division of Biology, Caltech, Pasadena, California 91125,; ^4^Department of Neurobiology and Anatomy, University of Utah, Salt Lake City, Utah 84132,; ^5^Rowland Institute at Harvard, Cambridge, Massachusetts 02142,; ^6^Department of Neuroscience, Yale University School of Medicine, New Haven, Connecticut 06510,; ^7^Center for Brain Science, Harvard University, Cambridge, Massachusetts 02138, and; ^8^Department of Molecular and Cellular Biology, Harvard University, Cambridge, Massachusetts 02138

**Keywords:** anatomy, asymmetry, motoneuron, reflex, vestibular, zebrafish

## Abstract

Within reflex circuits, specific anatomical projections allow central neurons to relay sensations to effectors that generate movements. A major challenge is to relate anatomical features of central neural populations, such as asymmetric connectivity, to the computations the populations perform. To address this problem, we mapped the anatomy, modeled the function, and discovered a new behavioral role for a genetically defined population of central vestibular neurons in rhombomeres 5–7 of larval zebrafish. First, we found that neurons within this central population project preferentially to motoneurons that move the eyes downward. Concordantly, when the entire population of asymmetrically projecting neurons was stimulated collectively, only downward eye rotations were observed, demonstrating a functional correlate of the anatomical bias. When these neurons are ablated, fish failed to rotate their eyes following either nose-up or nose-down body tilts. This asymmetrically projecting central population thus participates in both upward and downward gaze stabilization. In addition to projecting to motoneurons, central vestibular neurons also receive direct sensory input from peripheral afferents. To infer whether asymmetric projections can facilitate sensory encoding or motor output, we modeled differentially projecting sets of central vestibular neurons. Whereas motor command strength was independent of projection allocation, asymmetric projections enabled more accurate representation of nose-up stimuli. The model shows how asymmetric connectivity could enhance the representation of imbalance during nose-up postures while preserving gaze stabilization performance. Finally, we found that central vestibular neurons were necessary for a vital behavior requiring maintenance of a nose-up posture: swim bladder inflation. These observations suggest that asymmetric connectivity in the vestibular system facilitates representation of ethologically relevant stimuli without compromising reflexive behavior.

**SIGNIFICANCE STATEMENT** Interneuron populations use specific anatomical projections to transform sensations into reflexive actions. Here we examined how the anatomical composition of a genetically defined population of balance interneurons in the larval zebrafish relates to the computations it performs. First, we found that the population of interneurons that stabilize gaze preferentially project to motoneurons that move the eyes downward. Next, we discovered through modeling that such projection patterns can enhance the encoding of nose-up sensations without compromising gaze stabilization. Finally, we found that loss of these interneurons impairs a vital behavior, swim bladder inflation, that relies on maintaining a nose-up posture. These observations suggest that anatomical specialization permits neural circuits to represent relevant features of the environment without compromising behavior.

## Introduction

Neural circuits use populations of interneurons to relay sensation to downstream effectors that in turn generate behavior. The anatomical composition of interneuron populations has provided insight into its function. For example, interneuron populations are often organized into maps composed of nonuniformly sized sets of neurons similarly sensitive to particular features (Kaas, 1997). Such visual topography in the thalamus (Connolly and Van Essen, 1984) and cortex (Daniel and Whitteridge, 1961) magnifies the input from the central visual field. This magnification is thought to underlie enhanced perceptual acuity (Duncan and Boynton, 2003). Preferential anatomical organization is thought to facilitate adaptive olfactory (Hansson and Stensmyr, 2011), visual (Barlow, 1981; Xu et al., 2006), somatosensory (Adrian, 1941; Catania and Remple, 2002), and auditory (Knudsen et al., 1987; Bendor and Wang, 2006) computations. However, little is known about how these anatomical asymmetries within populations of sensory interneurons determine the activity of their target motor effectors. Motor anatomy shares a similar uneven organization (Kuypers, 2011), but the complex spatiotemporal encoding of muscle synergies has made comparable dissection of motor circuits more challenging (Levine et al., 2012; Shenoy et al., 2013; Harrison and Murphy, 2014). Even where descending cortical (Lemon, 2008) or brainstem (Esposito et al., 2014) neurons synapse directly on motoneurons, the complexity of most behaviors makes it difficult to relate anatomy to function. Data relating the anatomical projections of interneuron populations to their function are needed to address this problem.

By virtue of their defined connectivity, interneurons within central reflex circuits offer the opportunity to explore the relationship between population-level anatomical properties and function in a simpler framework. Vestibular interneurons, an ancient and highly conserved population, transform body/head destabilization into commands for compensatory behaviors, such as posture and gaze stabilization (Szentágothai, 1964; Straka and Baker, 2013; Straka et al., 2014). Gaze-stabilizing vestibular brainstem neurons receive innervation from peripheral balance afferents (Uchino et al., 2001) and use highly stereotyped axonal projections to particular extraocular motoneuron targets that produce directionally specific eye movements (Uchino et al., 1982; McCrea et al., 1987; Iwamoto et al., 1990b). One anatomical and physiological characterization of up/down-sensitive vestibular neurons in the cat suggested a potential 3:1 bias toward neurons responsible for downward eye movements (Iwamoto et al., 1990a) However, extracellular recording experiments may be subject to selection bias. Further, as selective activation has been impossible, whether there are functional correlates of putative anatomical specialization remains unknown.

To study the relationship between the anatomical specializations of interneuron populations and their functions, we investigated a genetically defined population of vestibular brainstem neurons in a model vertebrate, the larval zebrafish. Larval zebrafish face well-defined challenges that necessitate control of body orientation in the vertical/pitch axis (i.e., nose-up/nose-down). First, larval zebrafish rely on vestibular sensation to guide upward swimming to the water's surface to gulp air and inflate their swim bladders, a vital organ necessary to maintain buoyancy (Goolish and Okutake, 1999; Riley and Moorman, 2000). Further, fish actively maintain a nose-up posture (Ehrlich and Schoppik, 2017), permitting them to efficiently maintain their position in the water column despite being slightly denser than their surroundings (Aleyev, 1977; Stewart and McHenry, 2010). Larval zebrafish use vestibular brainstem neurons to stabilize gaze by performing torsional and vertical eye movements (Bianco et al., 2012). These same neurons project to nuclei responsible for movement initiation and pitch tilts (Pavlova and Deliagina, 2002; Severi et al., 2014; Thiele et al., 2014; Wang and McLean, 2014).

We leveraged known properties of the gaze stabilization circuit to relate the anatomy of a genetically defined population of vestibular brainstem neurons and their function. Our study reports three major findings. First, we discovered that central vestibular neurons in rhombomeres 5–7 (r5–r7) project preferentially to extraocular motoneurons that move the eyes down. Ablation of these neurons eliminates counter-rotation of the eyes following body tilts, establishing a role in gaze stabilization. Second, modeling revealed that asymmetrically projecting neurons could enhance the capacity to represent nose-up stimuli without compromising gaze stabilization. Third, we discovered that fish do not inflate their swim bladders following ablation of these interneurons. Together, our data suggest that the anatomical specialization we observe permits sensory specialization while maintaining reflexive capabilities.

## Materials and Methods

### 

#### 

##### Fish care.

All protocols and procedures involving zebrafish were approved by the Harvard University Faculty of Arts and Sciences Standing Committee on the Use of Animals in Research and Teaching (Institutional Animal Care and Use Committee). All larvae were raised at 28.5°C, on a standard 14/10 h light/dark cycle at a density of no more than 20–50 fish in 25–40 ml of buffered E3 (1 mm HEPES added). When possible, experiments were done on the *mitfa*^−/−^ background to remove pigment; alternatively, 0.003% phenylthiourea was added to the medium from 24 hpf onwards and changed daily. Larvae were used from 2 d post fertilization (dpf) to 11 dpf. During this time, zebrafish larvae have not determined their sex.

##### Behavior.

Torsional eye movements were measured following step tilts delivered using an apparatus similar in design to Bianco et al. (2012). All experiments took place in the dark. Larval fish were immobilized completely in 2% low-melting temperature agar (Thermo Fisher 16520), and the left eye was freed. The agar was then pinned (0.1 mm stainless minutien pins, FST) to a 5 mm^2^ piece of Sylgard 184 (Dow Corning), which was itself pinned to Sylgard 184 at the bottom of a 10 mm^2^ optical glass cuvette (Azzota, via Amazon). The cuvette was filled with 1 ml of E3 and placed in a custom holder on a 5-axis (X, Y, Z, pitch, roll) manipulator (ThorLabs MT3 and GN2). The fish was aligned with the optical axes of two orthogonally placed cameras such that both the left utricle and two eyes were level with the horizon (front camera).

The eye-monitoring camera (Guppy Pro 2 F-031, Allied Vision Technologies) used a 5× objective (Olympus MPLN, 0.1 NA) and custom image-forming optics to create a 100 × 100 pixel image of the left eye of the fish (6 μm/pixel), acquired at 200 Hz. The image was processed online by custom pattern matching software to derive an estimate of torsional angle (LabView, National Instruments), and data were analyzed using custom MATLAB scripts (The MathWorks). A stepper motor (Oriental Motors AR98MA-N5–3) was used to rotate the platform holding the cameras and fish. The platform velocity and acceleration were measured using integrated circuits (IDG500, Invensense and ADXL335, Analog Devices) mounted together on a breakout board (Sparkfun SEN-09268). Fish were rotated stepwise for 10 cycles: from 0° to −60°, where positive values are nose-down, then from −60° to 60°, and then back to 0° in 10° increments, with a peak velocity of 35°/s. The interstep interval was 5 s, and the direction of rotation was then reversed for the next sequence of steps.

The eye's response across the experiment was first centered to remove any offset introduced by the pattern-matching algorithm. Data were then interpolated with a cubic spline interpolation to correct for occasional transient slowdowns (i.e., missed frames) introduced by the pattern-matching algorithm. The eye's velocity was estimated by differentiating the position trace; high-frequency noise was minimized using a 4-pole low-pass Butterworth filter (cutoff = 3 Hz). Each step response was evaluated manually; trials with rapid deviations in eye position indicative of horizontal saccades or gross failure of the pattern-matching algorithm were excluded from analysis. The response to each step for a given fish was defined as the mean across all responses to that step across cycles. The gain was estimated by measuring the peak eye velocity occurring over the period 375–1000 ms after the start of the step. The steady-state response was estimated by measuring the mean eye position over the final 2 s of the step; the range was the difference between the most eccentric nose-up and nose-down steady-state angles.

Gain was evaluated over the range from 30° to −30° (i.e., the first three steps away from the horizontal meridian). We chose this interval for three reasons: (1) Fish spend the overwhelming majority of their time with a body orientation in this range (Ehrlich and Schoppik, 2017). (2) The responses here were the strongest, allowing us confidence in the dynamic capacity of the system without encountering the biophysical limits imposed by orbital structure. (3) Because the utricle conveys information both about static and dynamic changes in orientation, the eyes adopt an increasingly eccentric rotation as the stimulus progresses, potentially constraining dynamic range.

##### Transgenic lines.

*Tg(*−*6.7FRhcrtR:gal4VP16)*:-6.7FRhcrtR was amplified the using a nested PCR strategy. First, a 6775 bp DNA fragment immediately upstream of the Fugu rubripes hcrtr2 start site was amplified from genomic DNA, using a high-fidelity polymerase (PfuUltra II Fusion, Stratagene) with primers 5′-AATCCAAATTCCCAGTGACG-3′ and 5′-CCAGATACTCGGCAAACAAA-3′, 56°C annealing temperature, 1:45 elongation time. The PCR product was TOPO cloned into a TA vector (Thermo Fisher). Using the resulting plasmid as a template, a 6732 bp fragment was amplified using primers 5′-AATCCAAATTCCCAGTGACG-3′ and 5′-CCAGATACTCGGCAAACAAA-3′ 55°C annealing temperature, 1:45 elongation and similarly TOPO cloned into a GATEWAY-compatible vector (PCR8/GW, Thermo Fisher). The resulting entry vector was recombined into a destination vector upstream of gal4-VP16, between Tol2 integration arms (Urasaki et al., 2006). Tg(UAS-E1b:Kaede)s1999t embryos were injected at the one-cell stage with 0.5 nl of 50 ng/μl plasmid and 35 ng/μl Tol2 transposase mRNA in water, and their progeny screened for fluorescence. One founder produced three fluorescent progeny; one survived. To identify transgenic fish without using a upstream activating sequence (UAS) reporter, potential carriers were genotyped using the following primers to generate a 592 bp product spanning the upstream Tol2 arm and the start of the Fugu sequence: 5′-CAATCCTGCAGTGCTGAAAA-3′ and 5′-TGATTCATCGTGGCACAAAT-3′ 57°C annealing temperature, 0:30 elongation time. The complete expression pattern has been described previously (Lacoste et al., 2015) and is part of the Z-brain atlas (Randlett et al., 2015).

*Tg(14*×*UAS-E1b:hChR2(H134R)-EYFP)*:hChR2(H134R)-EYFP (Zhang et al., 2007) was subcloned downstream of 14 copies of a UAS element and an E1b minimal promoter in a vector containing an SV40 polyA sequence and Tol2 recognition arms (Urasaki et al., 2006). This vector was coinjected with tol2 transposase mRNA into TLAB embryos at the single-cell stage. Potential founders were screened by crossing to *Tg(isl1:Gal4-VP16,14*×*UAS:Kaede)* (Pan et al., 2012) and monitoring tail movements in response to blue light from an arc lamp on a stereomicroscope (Leica MZ16) at 30 hpf.

The following transgenic lines were used: *Tg(UAS-E1b:Kaede)^s1999t^* (Scott et al., 2007), *Tg(isl1:GFP)* (Higashijima et al., 2000), *Tg(UAS:KillerRed)* (Del Bene et al., 2010), *Tg(UAS-E1b:Eco.NfsB-mCherry)* (Pisharath et al., 2007), *atoh7^th241/th241^* (Kay et al., 2001), *Tg(atoh7:gap43-RFP)* (Zolessi et al., 2006), *Tg(5*×*UAS:sypb-GCaMP3)* (Nikolaou et al., 2012), and *Et(E1b:Gal4-VP16)^s1101t^* (Scott et al., 2007).

##### Anatomy.

To generate mosaically labeled fish, 0.5 nl of 30 ng/μl plasmid DNA *(14*×*UAS-E1b:hChR2(H134R)-EYFP)* (Douglass et al., 2008) or UAS-Zebrabow (Pan et al., 2013) was injected in water at the one-cell stage into *Tg(*−*6.7FRhcrtR:gal4VP16); Tg(isl1:GFP)* fish. Embryos were screened at 24–48 hpf. The majority (80%) of injected fish were excluded due to deformities or developmental arrest. The remaining fish were screened at 72 hpf under a fluorescent stereoscope (Leica MZ16) with a high-pass GFP emission filter for YFP fluorescence or a Cy3 emission filter for dTomato. As *Tg(*−*6.7FRhcrtR:gal4VP16)* will label the skin and notochord early (36–48 hpf), and fluorescence in either structure is relatively easy to visualize, embryos with mosaic labeling (usually 1–10 cells) in these structures were selected. On average, 1%–2% of injected embryos were retained for high-resolution screening. Larvae were anesthetized (0.016% w/v tricaine methane sulfonate, Sigma A5040), mounted dorsally at 5–7 dpf, and imaged on a confocal microscope (Zeiss 510, 710, or 780, using either a 20× 1.0 NA, a 40× 1.1 NA, or a 63× 1.0 NA objective with Zen 2010, 8-bit acquisition) with excitation of 488 nm (GFP) and 514 nm (EYFP), and emission for the two channels was either separated at 550 nm by a glass dichroic filters or a tunable filter. The two channels could reliably be separated provided the level of EYFP was strong relative to GFP.

**Figure 1.**
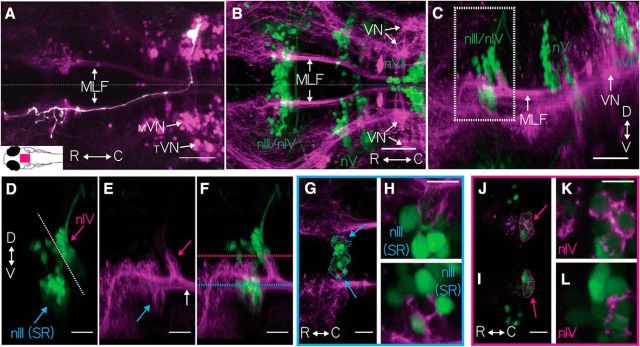
Vestibular nucleus neurons labeled in *Tg(*−*6.7FRhcrtR:gal4VP16)*. ***A***, The expression pattern of *Tg(*−*6.7FRhcrtR:gal4VP16); Tg(UAS-E1b:Kaede)s1999t* (purple) is shown as a horizontal MIP, with one vestibular neuron, colabeled by focal electroporation of *gap43-EGFP* (white). Arrows point to the tangential (_T_VN) and medial vestibular nuclei (_M_VN) and the MLF. Inset, Schematic of a dorsal view of a larval zebrafish. Magenta rectangle represents the location of the image. Scale bar, 50 μm. Horizontal (***B***) and sagittal (***C***) MIP of vestibular neurons in *Tg(*−*6.7FRhcrtR:gal4VP16);Tg(UAS-KillerRed)* (purple);*Tg(isl1:GFP)* (green, image γ = 0.5) showing cranial motoneuron somata from nIII/nIV, nV, and nVII (green text). Arrows indicate neurons in the vestibular nuclei (VN) and the MLF. Scale bar, 50 μm. ***D–F***, Close-up of white boxed region in ***C***, showing major branch patterns of vestibular neuron axon fascicle (purple) relative to extraocular motoneurons (green). ***D***, Motoneurons from *Tg(isl1:GFP)* (green) in nIV (magenta arrow), superior rectus motoneurons of nIII (cyan arrow), and the midbrain/hindbrain boundary (white dotted line). ***E***, Branches of the vestibular neuron axon fascicle (purple), emerging from the MLF (white arrow) in *Tg(*−*6.7FRhcrtR:gal4VP16);Tg(UAS-KillerRed)*, projecting to nIV (magenta arrow) and nIII (cyan arrow). ***F***, Merge of ***D*** and ***E***. Scale bar, 20 μm. ***G–I***, Broad and close-up views of vestibular neuron axonal projection (purple) to nIII cell bodies (green), taken at the horizontal plane delineated by the cyan dotted line in ***F***, SR motoneurons (nIII) encircled in cyan. ***G***, Cyan arrows localize close-ups in ***H*** and ***I***. Scale bar, 10 μm. ***J–L***, Broad and close-up view of vestibular neuron axonal projection (purple) to nIV cell bodies (green), taken at the horizontal plane delineated by the magenta dotted line in ***F***, SO motoneurons (nIV, green) encircled in magenta. ***J***, Arrows point to close-up in ***K*** and ***L***. Scale bar, 10 μm.

**Figure 2.**
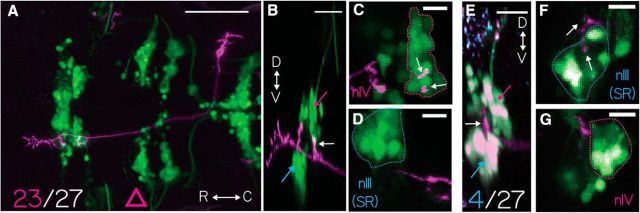
Projections from singly labeled vestibular nucleus neurons. ***A***, Horizontal MIP of a single vestibular neuron labeled with *UAS-ChR2(H134R)-EYFP* (purple) in *Tg(*−*6.7FRhcrtR:gal4VP16);Tg(isl:GFP)* (green). γ = 0.5 to highlight the sparse label. Scale bar, 100 μm. Pink triangle represents the data in Figure 7*D*. Twenty-three of 27 neurons studied projected similarly. ***B***, Sagittal MIP of the neuron in ***A*** highlighting nIII (cyan arrow), nIV (magenta arrow), and projection to nIV (white arrow). Scale bar, 20 μm. ***C***, Horizontal MIP of nIV (green cell bodies in dotted magenta outline) from ***A***. Vestibular neuron projection (purple, white arrow). Scale bar, 10 μm. ***D***, Horizontal MIP of nIII (green cell bodies in dotted cyan outline) with no proximal vestibular neuron projection (purple). ***E***, Sagittal MIP of a single axon expressing *14*×*UAS-E1b:hChR2(H134R)-EYFP* (purple) in *Tg(*−*6.7FRhcrtR:gal4VP16);Tg(isl1:GFP)* (green); *Tg(atoh7:gap43-RFP)* (cyan) fish. Expression of bright GFP bleeds into the purple channel, making the cell bodies white. nIV (magenta arrow), nIII (cyan arrow), and the vestibular neuron projection to SR motoneurons in nIII (white arrow). Scale bar, 20 μm. Four of 27 neurons projected similarly, exclusively to nIII. ***F***, Horizontal MIP of nIII (cells in blue outline) from ***E***, purple projections from vestibular neuron (white arrow). Scale bar, 10 μm. ***G***, Horizontal MIP of nIV (cells in magenta outline) from ***E*** with no purple vestibular neuron projection. Scale bar, 10 μm.

Most of the fish selected for confocal imaging had some neurons labeled in the brain, but on average, only 0.5%–2% (i.e., 5–20 for every 1000) of injected embryos would have vestibular nucleus neurons that were both bright and sufficiently isolated enough to trace. Neurons were only included in the study if their axon could be traced unambiguously throughout its entirety to a distinct cell body; qualitatively, the asymmetry persisted among excluded fish. Neurons were traced manually with the assistance of the ImageJ plugin Simple Neurite Tracer (Longair et al., 2011). Cell bodies of the oculomotor and trochlear nuclei were localized manually using the Fiji/ImageJ ROI functionality (Schindelin et al., 2012). Superior oblique motoneurons were found in nIV and superior rectus motoneurons were the most ventral somata in nIII (Greaney et al., 2017). All images were adjusted linearly, using the Brightness and Contrast functionality in Fiji/ImageJ (Schindelin et al., 2012). For display purposes, a nonlinear histogram adjustment (γ = 0.5) was applied to the maximum intensity projection (MIP) in Figures 1*B* and Figure 2*A* to increase the relative brightness of thin axonal arbors, and, for Figure 2*A*, to make clear the sparse nature of the label.

Retrograde labeling of the ocular motor nuclei was done as previously described (Ma et al., 2010; Greaney et al., 2017). In brief, crystals of fluorescently conjugated dextrans (10,000 molecular weight, Thermo Fisher D-1824 or D-22914) were placed in the left orbit of anesthetized 5–7 dpf fish. In fish, the superior eye muscles receive projections from the contralateral motor nuclei, making the relevant neurons in nIV (superior oblique) and nIII (superior rectus) easy to discriminate, as they were exclusively labeled on the contralateral (right) side.

Focal electroporations were done as detailed previously (Tawk et al., 2009; Bianco et al., 2012). Briefly, anesthetized larvae (2 dpf) were immobilized in low-melting temperature agarose. Micropipettes (tip diameter of 1–2 mm) were filled with a solution containing 1 mg/ml *gap43-EGFP* plasmid DNA in distilled water. To target the vestibular nucleus neurons, the pipette was placed at the lateral limit of rhombomere 5, using the decussation of the Mauthner axon midline crossing as a landmark. A Grass SD9 stimulator (Grass Technologies) was used to deliver three trains of voltage pulses in succession, with 1 s interval between trains. Each train was delivered at 200 Hz for 250 ms, 2 ms on time, with an amplitude of 30 V. Larvae were imaged at 5 dpf on a custom multiphoton microscope at 790 nm.

##### Lesions.

Single-cell ablations were performed using a pulsed infrared laser (SpectraPhysics MaiTai HP) at 820 nm (80 MHz repetition rate, 80 fs pulse duration) at full power: 200 mW (2.5 nJ) measured at the specimen with a power meter (ThorLabs S130C). Fish were mounted dorsally in 2% low-melt agarose in E3 under a 20× 0.95 NA objective (Olympus) and anesthetized as described above. Cell bodies were targeted for ablation based on anatomical location, starting with the most ventrolateral neurons in the tangential nucleus and then moving dorsomedially through the tangential and medial vestibular nucleus. Each cell was exposed to the pulsed infrared laser light for a brief period of time (35–50 ms), whereas the resulting fluorescent emissions were measured; usually, there was a brief pulse of light that saturated the detection optics which was used to shutter the laser. Five to 10 neurons/plane were targeted bilaterally, resulting in either loss of fluorescence (*Tg(UAS-E1b:Kaede)s1999t and Tg(isl1:GFP)*) or increased diffuse fluorescence at the cell body (*Tg(UAS-ChR2-E134R-EYFP)*). Fish were imaged immediately and 24 h after ablation to confirm the extent of the lesion; 15% of lesioned fish were excluded because they did not survive a full 24 h after the lesion. Fish were observed under a stereomicroscope in a Petri dish after lesion to ensure the presence of spontaneous horizontal saccades and normal jaw movements; all lesioned fish showed both. Fish for lesions were 4–5 dpf, as preliminary experiments showed that plasma formation was more effective in younger fish, and were selected to be the brightest in the clutch (likely doubly homozygous for *UAS-E1b:Kaede* and −6.7FRhcrtR:gal4VP16).

As previously described (Bianco et al., 2012), the eye movements in younger fish are of lower gain, and 3 of 17 fish were excluded from analysis because their total range was <10°. Behavior was always measured at least 4 h and no more than 8 h after lesions. The decrease in gain was reported as a percentage of prelesion gain, defined as the difference between the median prelesion gain and median after lesion gain normalized by the median prelesion gain. To activate *KillerRed*, green light (Zeiss set 43, 545 nm/25) from an arc lamp was focused through a 63 1.0 NA objective stopped down to fill a 200 μm diameter region for 15 min. Fish were mounted dorsally and anesthetized as described above. The focal plane was at the level of the decussation of the Mauthner axons, measured under brightfield illumination. Because of equipment replacement, the precise power of the arc lamp could not be measured, but 20 min of exposure under identical conditions was fatal to the fish. Postlesion behavior was measured at least 4 h after the light exposure. To induce apoptosis with nitroreductase, fish were placed in E3 with 7.5 mm of metrodinazole (Sigma M1547) in 0.2% v/v DMSO and behavior was measured 24 h later (Curado et al., 2007). The presence of mCherry fluorescence was assayed after behavior to determine genotype.

##### Optical activation and analysis.

Channelrhodopsin-induced eye movements were monitored using the same apparatus used for measuring tilt-induced behavior, with the addition of a fiber-coupled laser on an independent micromanipulator (Arrenberg et al., 2009; Schoonheim et al., 2010). Fish were immobilized and mounted as before, and agar was removed above the head as well as the left eye. Stimulus was generated by a 100 mW 473 nm diode laser (Shanghai DreamLasers SDL-473–100MFL) coupled by the manufacturer to a 50 μm inner diameter 0.22 NA multimode fiber (ThorLabs AFS50/125Y) that itself was butt-coupled to a 10 mm cannula made from the same diameter fiber (ThorLabs AFS50/125YCANNULA). Power at the cannula tip was 30–60 mW, measured with a power meter (ThorLabs S130C). The fiber tip was placed above the ear, evenly centered between the eyes, and 1 mm above the skin of the fish. Stimuli ranged in duration from 1 μs to 100 ms and were presented every 5 s. Eye movements were tracked and processed as before, including manual analysis; only fish with at least 25 analyzable responses to a given stimulus were included in the analysis. The response to a given stimulus was quantified by taking the peak angular rotation reached over the first 2 s.

By microinjecting plasmid DNA at the single-cell stage, we generated embryos as above with somatic expression of *ChR2-EYFP* in random subsets of vestibular neurons, on a blind background, *atoh7^th241/th241^* (Kay et al., 2001). As with anatomical experiments, between 5 and 20 fish for each 1000 injected had acceptable expression. Of these, only 1 of 4 were homozygous for *atoh7^th241^*, and only 1 of 4 of those expressed the allele necessary to confirm blindness by visualizing the absence of retinal ganglion cell axons *Tg(atoh7:gap43-RFP)*. The large number of alleles required and the low success rate limited the number of fish available to test. Tracing individual axonal projections to quantify the absolute number of VNs labeled in a given fish was not possible, except in the most sparsely labeled fish. Further, as expected with somatic expression, ChR2-EYFP levels varied considerably across vestibular neurons. To measure the relationship between expression levels/number of labeled neurons and the magnitude of the evoked eye movement, we quantified EYFP fluorescence. Vestibular neurons are the only neurons with rostral medial longitudinal fasciculus (MLF) projections labeled in *Tg(*−*6.7FRhcrtR:gal4VP16)*. As such, the total intensity of the MLF projection for a given fish was measured from the rostral-most point behind nIV, stopping caudally where the projection narrows to the midline (rhombomere 4). A single image that summed the intensity of all slices in the confocal stack that contained the MLF projection was used for our measurements. To correct for differences in acquisition parameters, MLF fluorescence was normalized by a measure of acquisition noise. Noise was estimated by measuring the summed fluorescence of a region between the branches of the MLF, which did not contain any neuropil. A value of 1 indicates no MLF fluorescence differentiable from background noise, a value of 2 indicates MLF fluorescence twice that of the background, etc. Ocular responses to blue light were evaluated and reported as above. Responses were evaluated for significance by comparing the median activity 200 ms after the stimulus to the baseline (200 ms before the stimulus).

##### Model.

Our model estimated the collective activity of 80 postsynaptic neurons generated by integrating activity from a set of presynaptic neurons. We evaluated two free parameters: the number of presynaptic neurons in the set (30, 42, 70, 105, 140, 168, 180) and the number of inputs on to a given postsynaptic neuron (2–30). Presynaptic activity was generated by translating a rate function, derived from the velocity profile of the steps used in the behavioral experiment, into a Poisson train of activity. Step velocity was scaled to match the reported velocity sensitivity (2 spikes/°/s) of second-order vestibular neurons (Iwamoto et al., 1990a) to generate a rate function for Poisson spikes. The velocity reached a peak of 35°/s and lasted 1 s; the model was run at 1 kHz. Poisson trains were subjected to an imposed 2 ms refractory period. The spikes were then convolved with a decaying exponential with τ = 1.5 s to represent an excitatory postsynaptic potential. A random subset of presynaptic neurons were selected from the set and summed together to create an input to a postsynaptic neuron. Postsynaptic activity was determined by thresholding the input, subject to a 2 ms refractory period. The threshold for the postsynaptic neuron was defined as the minimum of an input of 1.8% or 95% of the cumulative distribution of presynaptic input strength. One input spike had a value of 1; after convolution, a threshold of 1.8 was reached if at least four spikes were present across all inputs over a 4 ms period. Changing the threshold ensured that the postsynaptic response would not saturate as the number of inputs increased; the specific threshold did not change the relationships we observed and is expected from the basic properties of extraocular motoneurons (Torres-Torrelo et al., 2012). We generated 80 distinct spike trains, reflecting the number of motoneurons in a given motoneuron pool (Greaney et al., 2017). The total postsynaptic response was defined as the average activity, evaluated where the rate function was positive. The strength of the relationship between the presynaptic rate function and the summed post-synaptic response was defined as the coefficient of determination.

##### Experimental design and statistical analysis.

As data were not normally distributed, expected values are reported as the median, variability as the median absolute deviation, and nonparametric tests of significance were used. Potential differences between groups (e.g., up tilts vs down) were evaluated using the Wilcoxon rank sum test, and the Wilcoxon signed rank test was used to test whether a distribution had a median different from zero (e.g., change in performance after lesion). Significance was determined at *p* < 0.05.

## Results

### A genetically defined population of brainstem neurons projects preferentially to extraocular motoneurons that move the eyes downward

We adopted a molecular approach to characterize a subset of vestibular brainstem neurons in the larval zebrafish. We used a transgenic line of zebrafish, *Tg(*−*6.7FRhcrtR:gal4VP16)* that drives expression of a transcription factor (Gal4) in a restricted subset of neurons, including those in r5–r7 (Lacoste et al., 2015; Randlett et al., 2015). When crossed with other transgenic lines that contain an upstream activating sequence (UAS), Gal4 induces selective expression of particular genes useful for visualization, and for chemical or light-mediated manipulation. We first crossed the *Tg(*−*6.7FRhcrtR:gal4VP16)* to the *Tg(UAS-E1b:Kaede)^s1999t^* line to selectively drive a red fluorescent protein. In addition, we performed these experiments on a transgenic background, *Tg(isl1:GFP)*, which constitutively labeled cranial motoneurons, including extraocular motoneurons, with GFP.

Within r5–r7 (delineated by the rostro-caudal extent of the facial nucleus; Fig. 1*B*), we observed expression in ∼200 neurons that, in aggregate, comprise a subset of two bilateral vestibular nuclei. The first was the previously characterized utricle signal recipient tangential nucleus (Bianco et al., 2012), located adjacent to the ear. The second was the medial vestibular nucleus (Highstein and Holstein, 2006) separated from the tangential nucleus by the lateral longitudinal fasciculus. Figure 1*A–C* shows the gross morphology of these neurons and their axonal projections to the extraocular motor nuclei. In aggregate, we observed that the axon bundle from these vestibular neurons crosses the midline, ascends rostrally along the MLF, and projects to extraocular motor nuclei nIII and nIV (Fig. 1*D–F*).

The utricular vestibulo-ocular reflex uses two independent “channels,” or defined neural pathways from peripheral sensation to motor output, to stabilize gaze following pitch and roll tilts. At the level of the extraocular motoneurons, in the larval zebrafish, the two channels are segregated along the dorsoventral axis. First, the ventral-most extraocular motoneurons in nIII project to the inferior oblique (IO) and superior rectus (SR) motoneurons. Together, IO/SR move the eyes up following nose-down pitch tilts. Second, the dorsal-most extraocular motoneurons in nIII project to the inferior rectus (IR), and the dorsally located nucleus nIV projects exclusively to the superior oblique (SO). Together, IR/SO move the eyes down following nose-up pitch tilts. The somatic organization of nIII and nIV is stable after 5 dpf (Greaney et al., 2017). Finally, previous electromyographic recordings demonstrates that the SR (nIII) and SO (nIV) muscles are exclusively active during either the nose-down or nose-up phase of pitch-tilts supporting the independence of the two channels (Favilla et al., 1983).

Complementarily, pitch-sensitive vestibular nucleus neurons split into two subtypes, each projecting to only one pair of extraocular motoneurons (Uchino et al., 1982). The first group arborizes exclusively in nIII, innervating IO/SR. The second arborizes in both nIII and nIV, innervating SO/IR. Because nIV is comprised only of extraocular motoneurons that innervate SO, a collateral projection to nIV differentiates vestibular interneurons that respond to nose-up pitch tilts from those that respond to nose-down.

To determine whether vestibular neurons labeled in *Tg(*−*6.7FRhcrtR:gal4VP16)* comprise both nose-up and nose-down subtypes, we examined their collective projections. We observed that their projection terminated near the ventral-most extraocular motoneurons in nIII (Fig. 1*G*, wide view; Fig. 1*H*,*I*, close-up). The second prominent projection from vestibular neurons goes to extraocular motoneurons in nIV (Fig. 1*J*, wide view; Fig. 1*K*,*L*, close-up). We conclude that the vestibular neurons labeled in r5–r7 in *Tg(*−*6.7FRhcrtR:gal4VP16)* are poised to respond during both nose-up and nose-down pitch tilts.

**Figure 3.**
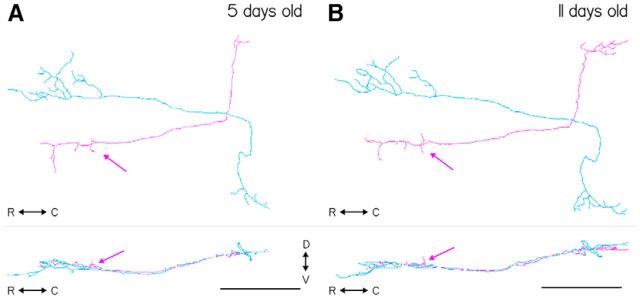
Tracings of two vestibular nucleus neurons from a single fish at two developmental time points. ***A***, Horizontal (top) and sagittal (bottom) projections of two traced neurons taken from the same fish imaged at 5 dpf. Magenta trace represents the characteristic projection to the nIV motoneuron pool (magenta arrows), whereas the green neuron does not. ***B***, Same two neurons traced in the same fish, at 11 dpf. The same projection to nIV is visible in the magenta tracing (magenta arrow). Scale bars, 100 μm.

To test whether the vestibular neurons labeled in *Tg(*−*6.7FRhcrtR:gal4VP16)* projected symmetrically to extraocular motoneurons, we examined the axon collaterals of singly labeled neurons. To differentiate nose-up from nose-down vestibular neurons, we manually traced the axons of vestibular neurons and used the labeled cranial motor nuclei to categorize their projections, based on the presence/absence of a collateral projection to nIV. We labeled stochastic subsets of vestibular neurons by injecting a plasmid encoding a fluorescent protein into one-cell embryos, *Tg(*−*6.7FRhcrtR:gal4VP16)*. Experiments were performed on the *Tg(isl1:GFP)* background to colabel extraocular motoneurons. The majority of labeled neurons (25 of 27) had only an ascending collateral; the remaining two had a bifurcated axon that both ascended and descended along the MLF. We found that the overwhelming majority (23 of 27) of labeled vestibular neuron axons had a dorsal collateral projecting to nIV (i.e., nose-up/eyes-down vestibular neurons). One example neuron from the majority population is shown projecting to nIV in Figure 2*A–D* and reconstructed as a schematic in Figure 2-1. In contrast, one example neuron from the minority population, projecting exclusively to nIII with a collateral to the superior rectus motoneurons, is shown in Figure 2*E–G* and reconstructed in Figure 2-2. Somata of neurons projecting exclusively to nIII were intermingled with those with projections to nIV. By examining labeled neurons at two time points (5 and 11 dpf) we found that the characteristic collateral projection to nIV in traced vestibular neurons remained unaltered (Fig. 3).

10.1523/JNEUROSCI.1711-17.2017.f2-1 Figure 2-1 Reconstruction of an nIV-projecting neuron. Data is taken from the same confocal stack shown in Figure 2A-2D. The neuron is shown in black, colored spheres represent the center locus of cell bodies of nIV (magenta) and nIII (cyan) cranial motoneurons. The movie begins with the neuron in a horizontal orientation, rotates 90° along the x axis until it is sagittal, and then rotates 90° along the y axis such that the viewer looks caudally down the long axis of the fish towards the tail. The large projection to nIV is clearly visible. Scale bar is 25 *µm* for all three axes. Download Figure 2-1, MOV file

10.1523/JNEUROSCI.1711-17.2017.f2-2 Figure 2-2 Reconstruction of an nIII-projecting neuron. Data is taken from the same confocal stack shown in Figure 2E-2G. The neuron is shown in black, colored spheres represent the center locus of cell bodies of nIV (magenta) and nIII (cyan) cranial motoneurons. The movie begins with the neuron in a horizontal orientation, rotates 90° along the x axis until it is sagittal, and then rotates 90° along the y axis such that the viewer looks caudally down the long axis of the fish towards the tail. The terminal arbors of this neuron are considerably more restricted than the neuron in M2, and bypass nIV, terminating in nIII. Scale bar is 25 *µm* for all three axes. Download Figure 2-2, MOV file

Our genetically based labeling technique is limited to neurons within the population labeled in *Tg(*−*6.7FRhcrtR:gal4VP16)*. To complement our initial characterization with an unbiased sample of vestibular neurons in r5–r7, we examined the projections of vestibular neurons that had been electroporated with a membrane-targeted fluorescent protein in wild-type animals. Of 20 electroporated animals with singly labeled neurons in the vestibular nuclei, 15 neurons had an ascending branch along the medial longitudinal fasciculus. Twelve of these (80%) had a prominent projection to nIV. Together, our data support the conclusion that vestibular neurons in the larval zebrafish project preferentially to extraocular motoneurons that move the eyes down.

**Figure 4.**
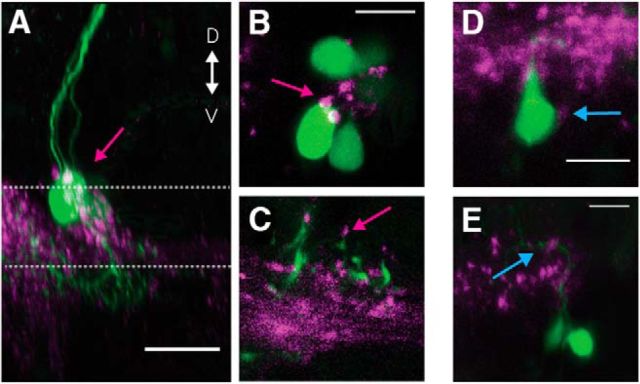
Vestibular nucleus neurons show synaptophysin-positive puncta on their motoneuron targets. ***A***, Sagittal MIP of a labeled SO motoneuron (magenta arrow) in green and the purple synaptic puncta labeled in Tg(−6.7FRhcrtR:gal4VP16); Tg(5×UAS:sypb-GCaMP3). Dotted lines indicate the planes in ***B***, ***C***. Scale bar, 20 μm. ***B***, ***C***, Close-up slice of the motoneuron somata in ***A*** with puncta (magenta arrow). Scale bar, 10 μm. ***D***, Close-up of a retrogradely labeled SR motoneuron soma (green) with visible purple puncta (cyan arrow). Scale bar, 10 μm. ***E***, Close-up of the dendrites of SR motoneurons (green) with visible purple puncta (cyan arrow). Scale bar, 10 μm.

To determine whether there was anatomical evidence that the axonal collaterals contained synapses, we labeled presynaptic puncta in *Tg(*−*6.7FRhcrtR:gal4VP16)* by crossing to *Tg(5*×*UAS:sypb-GCaMP3)* to selectively express a fluorescent protein fused to the presynaptic protein synaptophysin (Nikolaou et al., 2012). We then labeled the extraocular motoneurons by retro-orbital dye fill. We confirmed the presence of presynaptic puncta proximal to the soma and dendrites of SO and SR motoneurons (Fig. 4). Recent expansion microscopy work together with anti-synaptotagmin2b staining confirmed the presence of synaptic puncta between vestibular neurons labeled in *Tg(*−*6.7FRhcrtR:gal4VP16)* and extraocular motoneuron somata and dendrites (L. Freifeld and E. Boyden, unpublished observations). These results suggest that the axon collaterals from vestibular neurons labeled in *Tg(*−*6.7FRhcrtR:gal4VP16)* likely contain functional synapses.

### Labeled vestibular neurons are collectively necessary for gaze stabilization following both nose-up and nose-down body rotations

**Figure 5.**
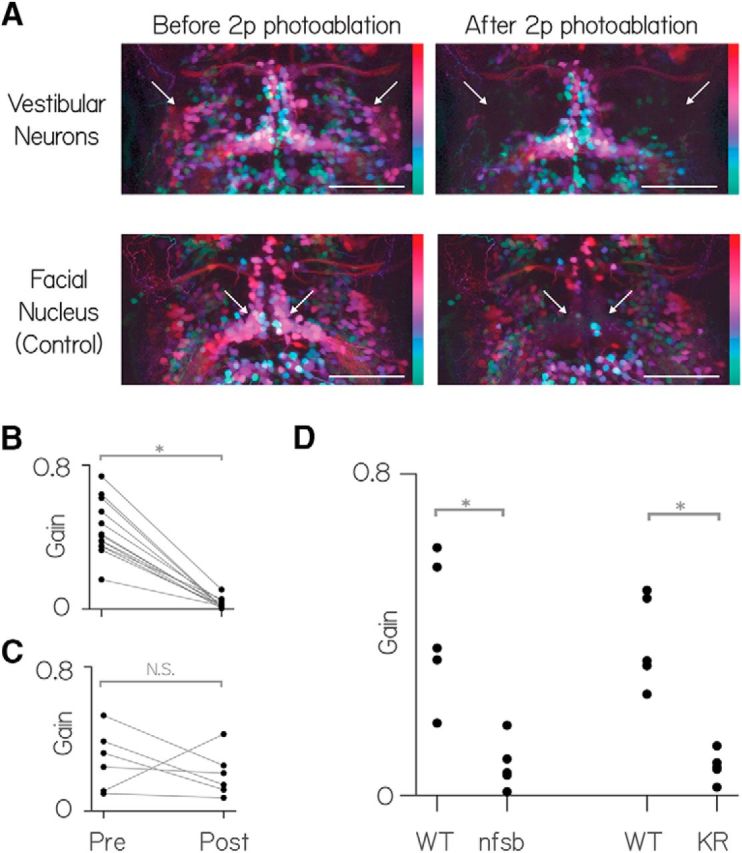
Vestibular nucleus neurons labeled in *Tg(*−*6.7FRhcrtR:gal4VP16)* are necessary for both nose-up and nose-down gaze stabilization. ***A***, Horizontal MIP of vestibular and control neurons (nVII) in rhombomeres 4–8 in *Tg(*−*6.7FRhcrtR:gal4VP16)*; *Tg(UAS-E1b:Kaede)^s1999t^*; *Tg(isl1:GFP)* fish before and after targeted photo-ablation of vestibular neuron cell bodies. γ = 0.5 highlights dim signal. Colors represent depth over ∼150 μm. White arrows indicate the general region of targeted cell bodies in either the vestibular nuclei (top row) or the facial nucleus (nVII). Scale bar, 150 μm. For anatomical localization, compare with the right side of Figure 1*B*. ***B***, Vestibulo-ocular reflex gain preablation and postablation of vestibular neurons. ***C***, Vestibulo-ocular reflex gain preablation and postablation of facial nucleus neurons. ***D***, Vestibulo-ocular reflex gain wild-type siblings (WT) and fish with pharmacogenetic (nitroreductase, “nfsb”) and optogenetic ablation (Killer-Red [KR]) of neurons in Tg(−6.7FRhcrtR:gal4VP16).

To determine whether the transgenically labeled vestibular neurons constitute a complete set necessary for both upward and downward eye movements following body tilts, we measured gaze stabilization (the vestibulo-ocular reflex) before and after their removal. We ablated single vestibular neurons individually with a pulsed infrared laser in *Tg(*−*6.7FRhcrtR:gal4VP16)*. These fish had been crossed to *Tg(UAS-E1b:Kaede)^s1999t^* to express a fluorescent protein in vestibular neurons. Further, experiments were performed on the Tg(isl1:GFP) background that labeled adjacent motoneurons in nVII for control ablations (Fig. 5*A*). Following ablation, qualitative observation revealed that horizontal eye saccades and spontaneous jaw movements were present as in normal fish. Ablations eliminated nearly the entire response to body tilts (both nose-up and nose-down): the median decrease in vestibulo-ocular reflex gain was 94.5 ± 3.5% (*n* = 14, *p* = 1.2 × 10^−4^; Fig. 5*B*).

We saw no difference (*p* = 0.77) in the postlesion gain for nose-up (0.0165 ± 0.0135) and nose-down (0.02 ± 0.0135) body rotations. In contrast, control lesions of somata in the adjacent facial nucleus (nVII) produced no systematic change in the gain (*n* = 5, 38.5 ± 24.5%, *p* = 0.41) or the range (31 ± 52%, *p* = 0.44; Fig. 5*C*) of the vestibulo-ocular reflex.

To confirm the finding that the labeled neurons in *Tg(*−*6.7FRhcrtR:gal4VP16)* were necessary for the normal vestibulo-ocular reflex following pitch tilts, we used two additional ablation techniques to target neurons labeled in *Tg(*−*6.7FRhcrtR:gal4VP16)*. First, by crossing to *Tg(UAS-E1b:Eco.NfsB-mCherry)*, we selectively expressed a protein, nitroreductase (nfsb) that caused neurons to die on exposure to a prodrug, metronidazole (Curado et al., 2007; Pisharath et al., 2007). After exposure to metronidazole, the vestibulo-ocular reflex was significantly impaired in larvae that expressed nfsb compared with their siblings that did not (*n* = 5, *p* = 0.008; Fig. 5*C*). Next, we crossed *Tg(*−*6.7FRhcrtR:gal4VP16)* to *Tg(UAS-KillerRed)* to selectively express a protein, Killer Red, that causes neurons to die on exposure to green light (Del Bene et al., 2010). After exposing the hindbrain to green light, the vestibulo-ocular reflex was significantly impaired in larvae that expressed Killer Red compared with similarly exposed siblings (*n* = 5, *p* = 0.008; Fig. 5*C*). We conclude that vestibular neurons labeled in *Tg(*−*6.7FRhcrtR:gal4VP16)* are necessary for compensatory eye movements following either nose-up or nose-down body pitch tilts.

### Labeled vestibular neurons, collectively activated, rotate the eyes down

**Figure 6.**
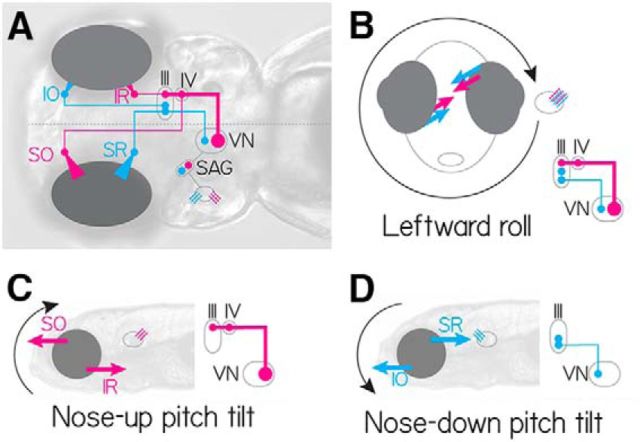
The simplified neural circuit underlying the ocular response to pitch and roll tilts. Cyan represents nose-down. Magenta represents nose-up channels. ***A***, Wiring diagram of one hemisphere of the excitatory vestibulo-ocular circuit showing utricular hair cells (cyan/magenta), stato-acoustic ganglion (SAG), central vestibular neurons (VN, cyan and magenta), extraocular motoneuron pools in nIII (SR, IR, IO) and nIV (SO). ***B***, During a roll tilt to the fish's left, the left utricle hair cells (cyan/magenta) are activated, causing cocontraction of superior (SO/SR) eye muscles ipsilateral to the activated utricle, and inferior (IO/IR) muscles contralateral to the activated utricle. ***C***, Utricular hair cells sensitive to nose-up pitch tilts (magenta) ultimately activate only vestibular neurons that project to both nIII and nIV, activating SO (contralateral) and IR (ipsilateral). ***D***, Utricular hair cells sensitive to nose-down pitch tilts (cyan) ultimately activate vestibular neurons that project to exclusively to nIII, activating SR (contralateral) and IO (ipsilateral).

The circuit that enables correct gaze stabilization following pitch and roll body tilts (Fig. 6) permits a specific prediction about the eye movements that might follow collective activation. Three key features of this circuit enable this prediction: (1) two distinct channels selectively sensitive to nose-up and nose-down rotations; (2) excitatory central neurons that all cross the midline; and (3) superior extraocular motoneurons that cross back. Figure 6*C*, *D* shows the torsional response to nose-up and nose-down body tilts. There, utricular hair cells in both the left and right ear sense the same pitch tilts. The projection patterns ensure that inputs from a given ear contacts the correct superior eye muscle on the ipsilateral side, and the correct inferior eye muscle on the contralateral side. In contrast, when the fish rolls, both nose-up and nose-down channels ipsilateral to the roll are activated. The two superior muscles are then activated ipsilaterally, whereas the two inferior muscles are activated contralaterally. In this way, a single circuit can respond appropriately to the two cardinal directions of body rotation sensed by the utricle, the sole source of vestibular sensation in young zebrafish (Beck et al., 2004; Mo et al., 2010; Bianco et al., 2012; Roberts et al., 2017).

Collectively activating all utricle signal recipient vestibular neurons is therefore equivalent to the fish rolling both leftward and rightward simultaneously. Consequentially, all four eye muscles on both sides would be expected to contract together. If no eye movement were to result, we would conclude that, despite the anatomical asymmetry, the nose-up and nose-down vestibular neuron pools were functionally equivalent. In contrast, a net downward rotation reflects stronger activation of the SO/IR motoneurons (nose-up, Fig. 6*C*) and weaker activation of the SR/IO motoneurons (nose-down, Fig. 6*D*). A net upward rotation reflects the opposite. Any vertical component (SO/SR vs IO/IR) to the eye movement would reflect uneven activation of neurons in the left versus right hemisphere (Fig. 6*B*) and would be dissociable from the torsional component. We hypothesized that the gaze stabilization circuit predicts that any systematic eye movement observed along the nose-up/nose-down axis following collective activation of vestibular brainstem neurons must reflect a functional bias in the set of activated neurons.

**Figure 7.**
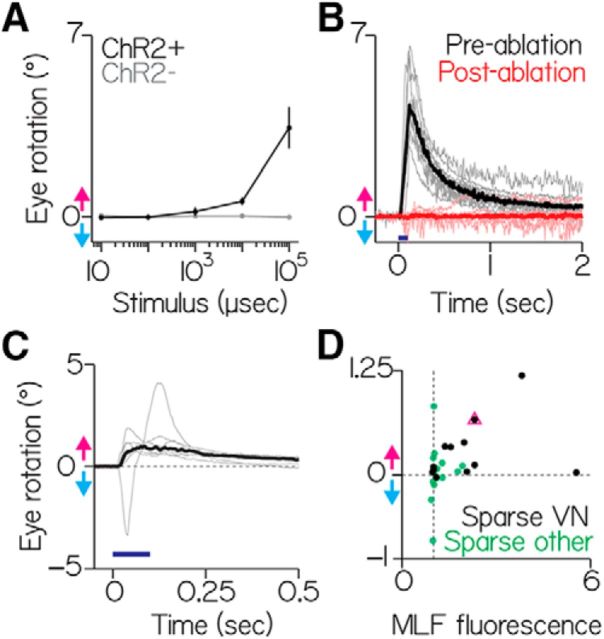
Activating vestibular nucleus neurons generates downward eye rotations. ***A***, Peak eye rotation as a function of blue light duration. Positive values indicate eyes-down rotations (magenta arrow). Negative values indicate eyes-up (cyan arrow). Black represents ChR2+ fish. Gray represents ChR2− siblings. Points are median ± median absolute deviation. ***B***, Evoked eye rotation in time. Gray lines indicate individual fish. Black lines indicate the median of prelesion data. Red lines indicate the same fish after photoablation of ChR2+ vestibular neurons. Blue represents stimulus (100 ms). ***C***, Gray lines indicate the average responses from individual fish with pan-neuronal expression. Black represents the median across fish. Blue represents stimulus (100 ms). The trace with a downward lobe indicates a nontorsional component; video of this fish is shown as Figures 7-1 and 7-2. ***D***, Evoked ocular rotations from sparsely labeled fish as a function of ChR2+ expression (MLF fluorescence). Black dots represent fish with discriminable vestibular neurons. Green dots represent fish without discriminable vestibular neurons. Pink triangle corresponds to the fish in Figure 2*A*.

To determine whether the asymmetry among the population of neurons we observed is functional, we measured eye rotations following collective activation of brainstem neurons labeled in *Tg(*−*6.7FRhcrtR:gal4VP16)*. We expressed the light-sensitive cation channel, channelrhodopsin-2 (ChR2) and used a fiberoptic cannula (Arrenberg et al., 2009) to target blue light to labeled vestibular neurons in *Tg(*−*6.7FRhcrtR:gal4VP16); Tg(UAS:ChR2(H134R)-EYFP)* fish. Because blue light evoked eye movements in wild-type fish, we performed all activation experiments using a blind mutant lacking retinal ganglion cells: *atoh7^th241/th241^*; *Tg(atoh7:gap43-RFP)* (Kay et al., 2001). Strikingly, in every transgenic fish tested, the eyes rotated downward in response to blue light flashes, as if the nose of the fish had moved up. We observed no systematic vertical component to the eye's rotation. Across fish (*n* = 10), the amplitude of eye rotation (Fig. 7*A*, black line) scaled with the duration of the light flash, with a peak response of 45°/s. Crucially, control siblings (*n* = 3) not expressing ChR2 did not respond to light flashes (Fig. 7*A*, gray line). Laser-mediated ablation of vestibular neurons abolished the light-evoked eye rotation (*n* = 10, Fig. 7*B*). Activation of the population of vestibular neurons is therefore sufficient to rotate the eyes downward, consistent with the asymmetric distribution of anatomical projections.

10.1523/JNEUROSCI.1711-17.2017.f7-1 Figure 7-1 A sample eye movement evoked by a blue light flash in fish expressing channelrhodopsin in vestibular neurons. The left eye of a Tg(-6.7FRhcrtR:gal4VP16); Tg(14xUAS-E1b:hChR2(H134R)-EYFP); atoh7th241/th241; Tg(atoh7:gap43-RFP) fish responding to a flash of blue light. Gray box reflects the realtime estimate of the eye’s rotation. Movie is 3.6sec long, with a 100msec flash at 1.2sec indicated by a cyan circle. Download Figure 7-1, MOV file

10.1523/JNEUROSCI.1711-17.2017.f7-2 Figure 7-2 A sample eye movement evoked in a fish with pan-neuronal channelrhodopsin. The left eye of a Tg(s1101t:gal4); Tg(14xUAS-E1b:hChR2(H134R)-EYFP); atoh7th241/th241; Tg(atoh7:gap43-RFP) fish responding to a flash of blue light. Green box reflects the realtime estimate of the eye’s rotation. Movie is 4 sec, with a 100ms flash after 2 sec. For clarity, the original video was enlarged 4x and slowed down 4-fold (200Hz acquisition, 50Hz playback). Note the rapid and transient change in the angle of the green square at the initiation of the evoked eye movement. This reflects the tracker failing due to the nasal-ward component of the eye’s contraction. After the brief failure of the tracker, it recovers, and the downward torsional component of the eye movement becomes visible. Download Figure 7-2, MOV file

We extended our test of sufficiency by activating all of the neurons in the region of the vestibular nucleus using a line reported (Scott et al., 2007) to drive expression in all neurons, *Et(E1b:Gal4-VP16)^s1101t^*. In all fish tested (*n* = 6), we evoked downward eye rotations in the torsional plane corresponding to nose-up tilts (Figs. 7*C*, 7-1, and 7-2, note the corruptive horizontal component present in one trace). Both genetically restricted and unbiased activation of vestibular neurons produced net downward eye rotations, and thus the gaze-stabilizing population of vestibular neurons is functionally asymmetric.

To test whether selective activation of vestibular neurons is sufficient to rotate the eyes, and to estimate the variability across neurons, we expressed ChR2 stochastically in subsets of neurons in *Tg(*−*6.7FRhcrtR:gal4VP16)* fish on a blind background *(atoh7^th241/th241^*; *Tg(atoh7:gap43-RFP)*. Of 27 sparsely labeled fish, 12 had expression in vestibular neurons. As expected from the uneven anatomy, all 12 had neurons with axon collaterals to nIV. Consistent with our categorization of nIV-projecting neurons as “nose-up/eyes-down,” we could evoke significant downward eye movements in 10 of 12 fish (0.23 ± 0.16°, *p* < 0.05 relative to baseline for each fish; Fig. 7*D*). Across all fish, the intensity of the projection in the MLF, an estimate of ChR2 expression, predicted the magnitude of the evoked response (Spearman's rank correlation coefficient = 0.45, *p* = 0.02, *n* = 27). These results reveal that subsets of nIV-projecting vestibular neurons are sufficient but vary in their ability to generate downward eye rotations.

### A simple model shows how biased vestibular populations can better represent nose-up sensations without compromising motor performance

Our data support the hypothesis that labeled premotor vestibular neurons are asymmetrically distributed, over-representing nose-up body tilts, and capable of producing downward eye rotations. To infer whether such an asymmetry might impact motor output and/or sensory encoding, we built a simple model of the synapse between vestibular and extraocular motoneurons. We simulated the ability of differently sized populations to relay a step-in body tilt (encoded by vestibular neuron activity) across a single synapse to produce an eye movement command (encoded by extraocular motoneuron activity). We constrained model parameters and assumptions to reflect known anatomical and electrophysiological properties (see Materials and Methods). For this model, we assume that the activity of the vestibular neurons is a function of body tilt. We systematically varied two free parameters: the size of the vestibular population and the number of vestibular neurons that contact a single extraocular motoneuron. As nose-up and nose-down neurons function during distinct phases of pitch tilts (Fig. 6), we simulated a single generic population. We evaluated two features of simulated motoneuron activity. First, as a measure of output strength, we report the average activity (reflecting the strength of ocular muscular contraction). Next, as a measure of encoding fidelity, we report the correlation between vestibular input and motoneuron output.

**Figure 8.**
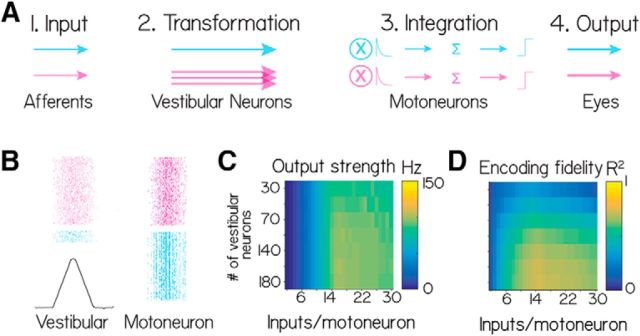
***A***, Model schematic. ***B***, One simulation of the model for two different population sizes, 180 neurons (magenta) and 30 neurons (cyan). First column represents the vestibular neuron activity as a spike raster plot and the input function (black). Second column represents the motoneuron spikes. For display, half the generated spikes are shown in each raster. ***C***, The “Output strength” (average firing rate) of the postsynaptic neurons as a function of the population size (rows) and number of inputs per motoneuron (columns). ***D***, The “Encoding fidelity” (variance explained, *R*) in the input rate function by the summed postsynaptic output.

We observed that the magnitude of motoneuron activity could be independent of the number of vestibular neurons upstream (Fig. 8*C*, vertical axis). This dissociation derives from the fact that vestibular neurons encoding nose-up and nose-down body rotations converge on to distinct pools of motoneurons. Consequentially, the key variable that determines the magnitude of motoneuron activity is the number of inputs per motoneuron, not the size of the vestibular population from which it is derived.

As expected, increasing the number of vestibular inputs onto a single motoneuron increased its firing rate asymptotically (Fig. 8*C*, horizontal axis). We conclude that when downstream effectors are distinct, as for eye movements, a larger pool of premotor neurons does not necessarily predict differences in the magnitude of motoneuron output. For our system, an asymmetric vestibular circuit could maintain comparable behavioral responses along the eyes-up/eyes-down axis.

In contrast, we observed that the size of the vestibular neuron pool could impact the ability of motoneurons to represent the dynamics of a step-in body position. Temporal structure emerges in the activity patterns of postsynaptic neurons derived from small population sizes (Fig. 8*B*). This similarity across motoneuron activity patterns reflected the coincidence of a limited set of inputs sufficient for a motoneuron spike at a particular time. To test whether this limitation constrains the ability of motoneurons to represent the input function, we measured the variance in the input rate function explained by 2 the summed motoneuron activity (*R*). Larger populations were indeed better than smaller populations, and performance varied with the precise number of presynaptic inputs (Fig. 8*D*). Adding a basal level of activity equal to 15% of the peak response decreased *R*^2^ but did not change the finding that larger populations were better at representing the input function. We infer from our model that the anatomical asymmetry we observe could permit better encoding of nose-up sensations without compromising gaze stabilization. If sensory statistics were similarly biased, asymmetric projections from vestibular neurons might therefore be adaptive.

### Premotor vestibular neurons are necessary for a vital and asymmetric postural behavior

To maintain buoyancy, larval zebrafish, whose gills do not yet function (Rombough, 2007), must swim to and maintain a nose-up posture at the water's surface, where they gulp air, inflating their swim bladder (Goolish and Okutake, 1999). Vestibular sensation is necessary: larval zebrafish without functional utricles fail to inflate their swim bladder and die (Riley and Moorman, 2000). In contrast, vision is not required for this behavior, as blind fish develop normal swim bladders. Gaze-stabilizing vestibular neurons send a second projection to a spinal premotor nucleus, the nucleus of the MLF (Fig. 1), indicating a potential postural role (Bianco et al., 2012).

**Figure 9.**
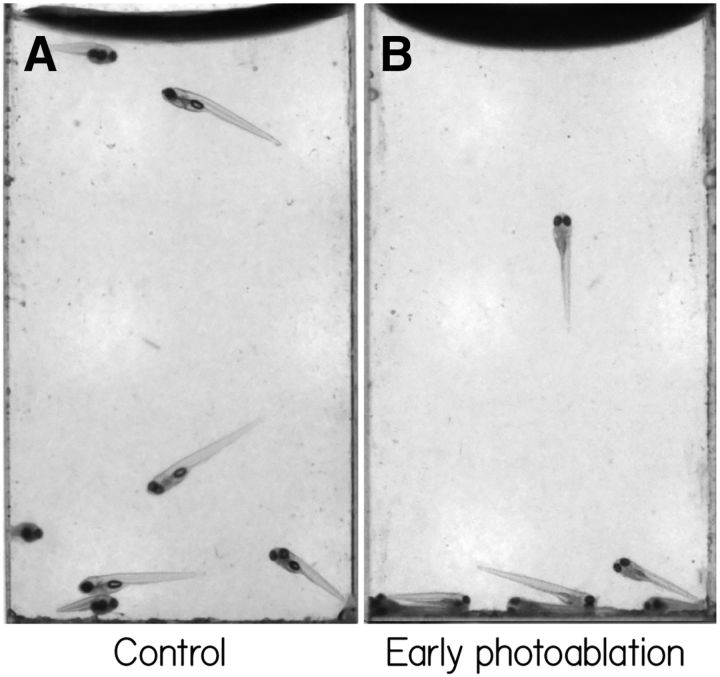
Early ablations of vestibular neurons leave fish unable to inflate their swim bladders. ***A***, Tg(−6.7FRhcrtR:gal4VP16); Tg(14×UAS-E1b:hChR2(H134R)-EYFP); mitfa^−/−^ fish swimming in a cuvette in the dark at 144 hpf. Red arrows point to swim bladders. ***B***, Sibling fish where the vestibular neurons in these fish were photoablated at 72 hpf, before swim bladder inflation. Note the absence of a swim bladder, evaluated here at 144 hpf. Images are taken from Figures 9-1 and 9-2.

To test whether vestibular neurons are necessary for swim-bladder inflation, we focally ablated vestibular neurons at 72 hpf, before fish had inflated their swim bladder, in *Tg(*−*6.7FRhcrtR:gal4VP16);Tg(14*×*UAS-E1b:hChR2(H134R)-EYFP)* fish. We evaluated the fish at 144 hpf (Fig. 9). Only 1 of 9 lesioned fish (example in Fig. 9-1) had an inflated swim bladder, compared with 40 of 42 control siblings (example in Fig. 9-2).

10.1523/JNEUROSCI.1711-17.2017.f9-1 Figure 9-1 Fish without vestibular neurons swimming in a cuvette. Tg(-6.7FRhcrtR:gal4VP16); Tg(14xUAS-E1b:hChR2(H134R)-EYFP); mitfa -/- fish swimming in a cuvette. Vestibular neurons in these fish were photoablated at 72hpf, before swim bladder inflation. Note the absence of a swim bladder, evaluated here at 144hpf. These fish are siblings of the fish in Movie M7. Download Figure 9-1, MOV file

10.1523/JNEUROSCI.1711-17.2017.f9-2 Figure 9-2 Fish swimming in a cuvette. Tg(-6.7FRhcrtR:gal4VP16); Tg(14xUAS-E1b:hChR2(H134R)-EYFP); mitfa -/- fish filmed at 144hpf. Note the presence of a swim bladder. These fish are siblings of the fish in Movie M6. Download Figure 9-2, MOV file

To confirm these results, we chemogenetically ablated vestibular neurons at 72 hpf in *Tg(*−*6.7FRhcrtR:gal4VP16);Tg(UAS-E1b:Eco.NfsB-mCherry)* fish. As with the targeted lesions, only 1 of 36 double-transgenic fish inflated their swim bladder and survived, whereas 36 of 36 of their nonexpressing siblings did. We note that, in contrast, fish with postinflation loss of vestibular neurons (e.g., Fig. 5) maintain normal swim bladders. These results define a novel role for vestibular neurons labeled in *Tg(*−*6.7FRhcrtR:gal4VP16)* in swim-bladder inflation.

## Discussion

We investigated how the anatomical composition of a genetically defined population of vestibular interneurons in the larval zebrafish could constrain its function. We first discovered that genetically labeled neurons project preferentially to motoneurons that move the eyes downward. Ablation of these neurons eliminated the eye movements normally observed following nose-up/nose-down body tilts, establishing their necessity for gaze stabilization. Next, we found that activation produced downward eye rotations, establishing a functional correlate of the anatomical asymmetry. We modeled similar populations with asymmetric projections and inferred that such architecture could permit better representation of nose-up stimuli while maintaining gaze stabilization performance. Finally, we discovered that early ablation of these neurons impaired swim bladder inflation, a vital postural task requiring nose-up stabilization. Together, we propose that preferential allocation of vestibular resources may improve sensory encoding, potentially enabling larval zebrafish to meet ethologically relevant challenges without compromising behavior.

Our study used a transgenic line, *Tg(*−*6.7FRhcrtR:gal4VP16)*, to reliably access a genetically defined set of neurons in rhombomeres 5–7 in the medial and tangential vestibular nuclei. The rhombomeric and mediolateral location of these neurons is consistent with the neurons that receive utricular input in the adult frog (Straka et al., 2003) and chick (Popratiloff and Peusner, 2007) and comprises a subset of neurons that project to extraocular motoneurons in the larval frog (Straka et al., 2001), juvenile zebrafish/goldfish (Suwa et al., 1996), and chick (Gottesman-Davis and Peusner, 2010). *Tg(*−*6.7FRhcrtR:gal4VP16)* does not label neurons within the superior vestibular nucleus in the rostral hindbrain (Cambronero and Puelles, 2000). This absence is notable in light of our ablation experiments that implicate only the neurons labeled in *Tg(*−*6.7FRhcrtR:gal4VP16)* as necessary for the torsional vestibulo-ocular reflex. Neurons in the superior vestibular nucleus receive input predominantly from the anterior canal and the lagena (Straka et al., 2003), and from the anterior canal in monkeys (Yamamoto et al., 1978). Larval zebrafish do not have functional semicircular canals (Beck et al., 2004), nor has the lagena developed (Bever and Fekete, 2002) at the ages we studied here. Therefore, superior vestibular nucleus neurons would not be expected to respond to body rotations, consistent with our observation that the eyes no longer counter-rotate after lesions of *Tg(*−*6.7FRhcrtR:gal4VP16)*-positive neurons. Further, the superior vestibular nucleus contains predominantly ipsilaterally projecting, likely inhibitory inputs in adult rays (Puzdrowski and Leonard, 1994), goldfish (Torres et al., 1992, 1995), frog (Montgomery, 1988), rabbit (Wentzel et al., 1995), cat (Carpenter and Cowie, 1985), and monkey (Steiger and Büttner-Ennever, 1979). In the adult goldfish, such inhibitory inputs to extraocular motoneurons were found to be less effective relative to their excitatory counterparts. If the vestibular circuit were similarly constrained in larval zebrafish, it could explain the smaller downward eye movement we saw after collective activation of all neurons. There, the normal downward eye rotation would be compromised, although not eliminated, by inhibition derived from superior vestibular nucleus neurons not labeled in *Tg(*−*6.7FRhcrtR:gal4VP16)* but activated in a pan-neuronal line. We therefore propose that the inputs and output of superior vestibular nucleus neurons not labeled in *Tg(*−*6.7FRhcrtR:gal4VP16)* render them unlikely to play a major role in the larval zebrafish torsional vestibulo-ocular reflex.

Previous work in larval zebrafish identified the tangential nucleus as the locus of neurons responsible for the utricle-dependent torsional vestibulo-ocular reflex (Bianco et al., 2012). Here, we show similarly profound impairment of the torsional vestibulo-ocular reflex after targeted ablation of a subset of vestibular interneurons in the tangential and medial vestibular nuclei that are labeled in *Tg(*−*6.7FRhcrtR:gal4VP16)* larvae. Therefore, we propose that the set of tangential nucleus neurons labeled in *Tg(*−*6.7FRhcrtR:gal4VP16)* are responsible for the utricle signal-mediated torsional vestibulo-ocular reflex, as those were ablated both here and in Bianco et al. (2012).

Similarly, previous single-cell fills of tangential nucleus neurons revealed three classes of projection neurons: those projecting to the contralateral tangential nucleus, those with a single ascending collateral to nIII/nIV and the nucleus of the MLF, and those with both an ascending and descending branch. Ascending and ascending/descending neurons were represented approximately equally (7 of 16 and 6 of 16) in the tangential nucleus (Bianco et al., 2012). However, we found that the labeled neurons in *Tg(*−*6.7FRhcrtR:gal4VP16)* were almost exclusively of the ascending type (25 of 27). We therefore propose a further refinement: the neurons responsible for the utricle-mediated torsional vestibulo-ocular reflex are likely the subpopulation of ascending neurons within the tangential nucleus labeled in *Tg(*−*6.7FRhcrtR:gal4VP16)*. This proposal is consistent with anatomical and functional work in juvenile and adult goldfish, where tangential nucleus neurons with ascending processes were shown to respond to nose-up/nose-down tilts (Suwa et al., 1999) Together, our molecular approach permits strong hypotheses that define the essential subset of vestibular neurons responsible for a particular behavior.

The muscles that generate torsional eye movements in fish are responsible for vertical eye movements in frontal-eyed animals (Simpson and Graf, 1981). The behavioral literature is unclear with respect to whether nose-up/nose down gaze stabilization is asymmetric. Cats may produce stronger downward eye rotations (Darlot et al., 1981; Tomko et al., 1988; Maruyama et al., 2004), but the literature is conflicted as to whether or not such an asymmetry exists in primates: downward (Benson and Guedry, 1971; Baloh et al., 1983; Matsuo and Cohen, 1984) or no biases (Baloh et al., 1986; Demer, 1992; Marti et al., 2006) have both been reported. In foveate vertebrates, the vestibular brainstem contains the final premotor nuclei for smooth pursuit eye movements. Despite similar abilities to perceive both directions of vertical motion (Churchland et al., 2003), both juvenile and mature monkeys (Grasse and Lisberger, 1992; Akao et al., 2007) and humans (Ke et al., 2013) show a stronger downward response. Our model points a way forward: while common laboratory stimuli may elicit largely similar vertical eye movements, an asymmetric population should better encode dynamic variability, such as experienced in natural settings (Carriot et al., 2014). We propose that characterizing the variation in response to more complex body rotations and target tracking paradigms could uncover behavioral signatures of an anatomically biased circuit.

We found that larval zebrafish do not inflate their swim bladders after early but not late ablation of vestibular neurons. As autonomic neurons are thought to determine swim bladder volume (Smith and Croll, 2011), we propose that the failure to inflate the swim bladder is secondary to postural impairments that follow loss of vestibular neurons labeled in *Tg(*−*6.7FRhcrtR:gal4VP16)*. In addition to extraocular motor nuclei, these neurons project to the nucleus of the medial longitudinal fasciculus (nMLF) (Bianco et al., 2012). Recent work has established the necessity and sufficiency of spinal-projecting neurons in the larval zebrafish nMLF for postural control and swim initiation (Severi et al., 2014; Thiele et al., 2014; Wang and McLean, 2014).

By virtue of their direct projections, and their necessity for swim bladder inflation, we propose that neurons labeled in *Tg(*−*6.7FRhcrtR:gal4VP16)* may affect posture by modulating activity of neurons in the nMLF. As such, our work thus establishes a new molecularly accessible avenue to explore neural mechanisms underlying postural stabilization.

Zebrafish engage in postural behaviors across their lifespan that are well suited to nose-up sensory specialization. First, as larvae, they swim along a trajectory dictated by the long axis of their body (Aleyev, 1977). Their bodies are denser than water (Stewart and McHenry, 2010), which ought to cause them to sink. Instead, they adopt a nose-up bias to their posture (Ehrlich and Schoppik, 2017), which introduces a vertical component to their swims, enabling them to maintain elevation. Second, larval zebrafish must swim to the surface to gulp air necessary to inflate their swim bladder (Goolish and Okutake, 1999). Finally, most adult teleosts engage in aquatic surface respiration throughout life (Kramer and McClure, 1982), a response to low oxygen saturation that necessitates a continuous nose-up posture at the water's surface. Our model shows how the anatomical makeup of the vestibular circuits could better encode the nose-up bias in the statistics of behavior. Our findings thus provide a premotor complement to the “efficient coding” framework used to relate the makeup of sensory systems to the statistics of the environment (Simoncelli, 2003).

Asymmetrically organized populations of interneurons are common throughout nervous systems. Asymmetric organization within sensory areas is thought to reflect afferent adaptations (Adrian, 1941; Barlow, 1981; Knudsen et al., 1987; Catania and Remple, 2002; Simoncelli, 2003; Bendor and Wang, 2006; Xu et al., 2006; Hansson and Stensmyr, 2011), but the complexity of most neural circuits makes it challenging to link encoding capacity to adaptive behavior. For asymmetric motor populations, links to behavior are more direct (Pasqualetti et al., 2007; Lemon, 2008; Rathelot and Strick, 2009; Esposito et al., 2014), but the natural sensations that drive these areas are often difficult to define. Our study of vestibular interneurons that play both sensory and premotor roles illustrates how the asymmetric anatomy could better encode nose-up sensations while maintaining the ability to stabilize gaze. As asymmetric populations of interneurons are common, we propose that other circuits may use similar strategies to meet ethological demands without compromising motor control.
